# A Stratified Acoustic Model Accounting for Phase Shifts for Underwater Acoustic Networks

**DOI:** 10.3390/s130506183

**Published:** 2013-05-13

**Authors:** Ping Wang, Lin Zhang, Victor O. K. Li

**Affiliations:** 1 Department of Electronic Engineering, Tsinghua University, Beijing 100084, China; E-Mail: ping-wang07@mails.tsinghua.edu.cn; 2 Department of Electrical and Electronic Engineering, the University of Hong Kong, Hong Kong; E-Mail: vli@eee.hku.hk

**Keywords:** transmission loss prediction, stratified acoustic model, geometrical ray tracing, phase shifts

## Abstract

Accurate acoustic channel models are critical for the study of underwater acoustic networks. Existing models include physics-based models and empirical approximation models. The former enjoy good accuracy, but incur heavy computational load, rendering them impractical in large networks. On the other hand, the latter are computationally inexpensive but inaccurate since they do not account for the complex effects of boundary reflection losses, the multi-path phenomenon and ray bending in the stratified ocean medium. In this paper, we propose a Stratified Acoustic Model (SAM) based on frequency-independent geometrical ray tracing, accounting for each ray's phase shift during the propagation. It is a feasible channel model for large scale underwater acoustic network simulation, allowing us to predict the transmission loss with much lower computational complexity than the traditional physics-based models. The accuracy of the model is validated via comparisons with the experimental measurements in two different oceans. Satisfactory agreements with the measurements and with other computationally intensive classical physics-based models are demonstrated.

## 1. Introduction

Underwater acoustic networks (UANs) are composed of underwater sensor nodes and underwater autonomous vehicles (UAV) that work in a collaborative manner to perform specific tasks, such as oceanographic data collection, pollution monitoring, offshore exploration, seismic/tsunami monitoring and prediction, tactical surveillance, and navigation [1]. The main difference of UANs from conventional terrestrial wireless sensor networks and wireless *ad hoc* networks stems from the complex underwater acoustic communication channel, in which the information carrier is the sound wave instead of the electromagnetic wave. Underwater acoustic communication is challenging and complex due to the varying speed of sound within the water medium and interactions at the sea surface and/or ocean floor, leading to complicated time-varying multi-path effects, and environment-dependent transmission losses. Therefore, an accurate and efficient model for the underwater acoustic channel is critical for the study of large scale underwater acoustic networks. In this paper, we aim to develop a stratified acoustic model (SAM) that has satisfactory accuracy and acceptable computational complexity for large scale underwater acoustic network simulations.

Efforts on modeling the sound propagation in the ocean medium date back to the 1920s, much earlier than the birth of the UANs [2–5]. These early research endeavors resulted in propagation models that later become the cornerstones for the contemporary underwater acoustic communication technologies. The most popular models are physics-based, and rely on the mathematical solutions to the reduced wave equation, also known as the Helmholtz equation. Different approaches to the solution of the Helmholtz equation result in different models, such as normal mode [6,7], parabolic equation [8,9], wavenumber integration (also called fast field programs) [10,11] and ray theory [12,13], with different working conditions. These models give accurate results at the expense of highly complex computations.

In studies of large scale underwater networks, researchers prefer to use the less accurate but computationally less expensive empirical channel models [13–18], which only account for the geometrical spreading loss and the absorption loss and are only dependent on the physical distance and the signal frequency. Although the empirical model remains the most successful and widely used channel model, it neglects the multi-path propagation and refraction effects caused by the ocean medium boundaries (*i.e.*, sea surface and sea bottom) and inhomogeneity, resulting in inaccurate propagation loss and delay estimations. This fact has been gaining recognition in the research community, leading to an increasing awareness on the needs for underwater channel models that are both accurate and computationally affordable.

Zielinski *et al.* developed a simple but effective model for multi-path channels, and used it to assess the performance of a digital communication system operating in shallow waters [19]. Chitre *et al.* developed a physics-based model for the very shallow warm-water acoustic channel at high frequencies, incorporating the time-varying statistical effects as well as non-Gaussian ambient noise statistics [20]. Abdi and Zajic studied the mathematical characterization of different types of correlations in acoustic vector sensor arrays statistically, using probabilistic models for the random components of the underwater propagation environment [21,22]. However, the layered structure of the ocean medium, which causes variant sound speeds at different depths and greatly impacts the propagation of sound in deep-sea environments, was ignored.

The main contribution of this work is a stratified acoustic model (SAM) for UANs. This model provides satisfactory accuracy while maintaining an acceptable computational complexity for large scale network simulations. SAM is validated with experimental measurements in two different oceans. Satisfactory agreements with the measurements and with other computationally intensive classical physics-based models are demonstrated.

The remainder of the paper is organized as follows. Section 2 introduces basic characteristics of the sound propagation in the ocean medium, and provides motivations for designing an improved underwater model for transmission loss prediction; Section 3 presents SAM for predicting transmission loss for underwater acoustic communication; Section 4 validates SAM through comparisons with two real-world measured data sets and with other channel models; Section 5 illustrates the application of SAM to study the impacts of the distribution of sound speed and the depths of the destination on the transmission loss; and Section 6 concludes with suggestions for future research.

## 2. Background and Motivation

### 2.1. Characteristics of Sound Propagation in the Ocean Medium

#### 2.1.1. Ray Bending in the Stratified Ocean Medium

**Figure 1.**
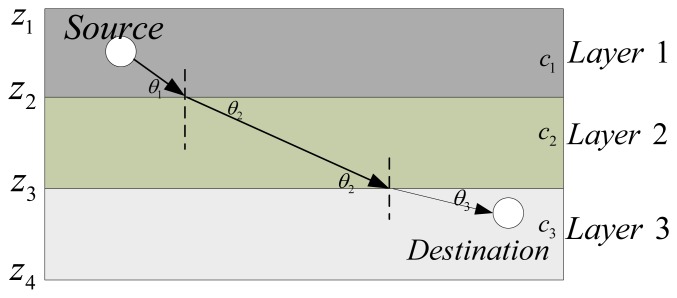
Sound ray propagation in stratified ocean medium.

The ocean is a heterogeneous medium, featuring water layers with different temperatures, increasing pressure with depth and location-dependent salinity [12]. All these factors lead to sound speed variations in the water. Amongst the three factors, pressure is the most significant one, and, in this paper, we assume that the sound speed profile (SSP) is horizontally stratified. In SAM, the ocean medium is modeled as *N* layers, with Layer *i* at a depth from *z_i_* to *z_i_*_+_*_1_* and the variation of sound speeds with depths can be expressed by a piecewise linear function as in [12,23,24]. Within each layer, the sound speed is approximated with a linear equation so that, within Layer *i*, at depth *z*, *z_i_* ≤ *z* ≤ *z_i+1_*, the sound speed is taken to be c(*z*) = *c_i_* + (*z*−*z_i_*)*g_i_*, where *c_i_* is the sound speed at depth *z_i_*, and *g_i_* is the sound speed gradient in the segment from *z_i_* to *z_i_*_+_*_1_*. An illustration of the sound propagation in the ocean with *N* = *3* and *g*_1_ = *g*_2_ = *g*_3_ = 0 is shown in Figure 1. According to Snell's Law [25], at the boundary of Layers 1, 2, and 3, the three grazing angles *θ*_1_, *θ*_2_, and *θ*_3_ are determined by:

(1) $$\frac{\cos\theta_{1}}{c_{1}}=\frac{\cos\theta_{2}}{c_{2}}=\frac{\cos\theta_{3}}{c_{3}}=\text{const}$$

where *c*_1_, *c*_2_, *c*_3_ and are the sound speeds at Layer 1, Layer 2, and Layer 3, and *c*_1_ < *c*_2_ < *c*_3_. Therefore, an acoustic ray bends away from the normal line of the boundary while the sound speed increases and towards the normal line while the sound speed decreases.

Since the sound rays do not propagate in straight lines, the amplitudes, phases and arrival times of rays will vary in different ocean environments. Moreover, the actual length of the sound trajectory between a pair of transceivers is not equal to the Euclidean distance. However, empirical computation does not take this into account. SAM will incorporate this stratification effect on predicting the transmission loss.

#### 2.1.2. Interactions of Sound Rays on the Boundaries

**Figure 2.**
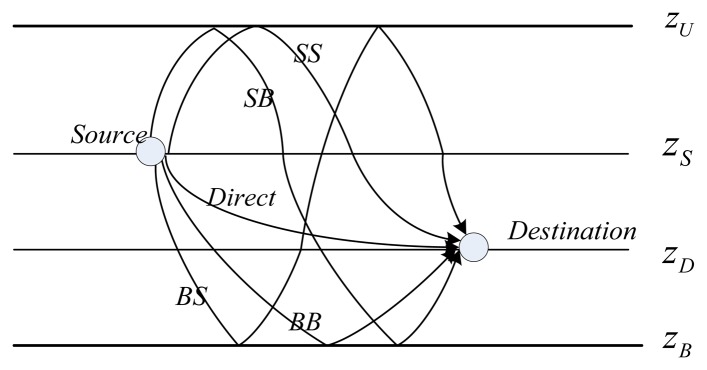
Multiple propagation paths for a pair of nodes.

Besides the ray bending in the heterogeneous ocean medium, reflections and refractions on the medium boundaries (*i.e.*, sea surface and sea floor), as shown in Figure 2, also have important effects on the propagation of the sound.

The impedance mismatch between the seawater and the air causes the sea surface to be a very good reflector. When a sound wave in water hits a smooth sea surface, nearly all of the incident energy is coherently reflected in the forward direction. However, as the surface becomes rougher, sound is also scattered in the backward and out-of-plane directions due to the surface irregularities. Considering that scattered waves in the backward and out-of-plane directions have little chance to reach the destination node located in the forward direction with sufficient power, the sea-air interface is considered as a perfect reflector [12]. While the phase shift experienced is stated as a “π phase shift”, the effect of the scattering is taken into account by assigning a reflection coefficient with a magnitude less than one.

There are many efforts on estimating surface loss due to the surface roughness [26–30]. Here the surface reflection coefficient is evaluated using, for instance, the Bechmann-Spezzichino model [29]. Therefore, the magnitude of the sea surface reflection coefficient is given by:

(2) $$\gamma_{s}=\sqrt{\frac{1+{\left(f/f_{1}\right)}^{2}}{1+{\left(f/f_{2}\right)}^{2}}}$$

where *f*_2_ = 378*ω*^−2^ and $f_{1}=\sqrt{10}f_{2}$. In Equation (2), *f* is the carrier frequency in kHz and *ω* is the wind speed in knots. Specifically, considering the π phase shift due to the reflection from the sea surface [29], the surface pressure complex reflection coefficient *γ_s_* is expressed by:

(3) $$\gamma_{s}=-\left|\gamma_{s}\right|$$

The sea floor is another reflecting and scattering boundary, having a number of characteristics similar to that of the sea surface. The bottom reflection can be described using the ocean bottom reflection coefficient *γ_b_* as [3,31–33]

(4) $$\gamma_{b}=\left|\gamma_{b}\right|e^{j\emptyset }$$

where |*γ_b_*| is the magnitude of bottom reflection coefficient and ø is the phase shift. According to Reference [3], $|\gamma_{b}|=\frac{v\sin\theta_{1}-\sqrt{\mu^{2}-{\left(\cos\theta_{1}\right)}^{2}}}{v\sin\theta_{1}+\sqrt{\mu^{2}-{\left(\cos\theta_{1}\right)}^{2}}}$ and $\emptyset =-2\tan^{-1}\frac{\sqrt{{\left(\sin\theta_{1}\right)}^{2}-\mu^{2}}}{v\cos\theta_{1}}$, where $\mu =\frac{c_{w}}{c_{b}}$ is the ratio of the water sound speed c_w_ to the bottom material sound speed c_b_, $\text{v}=\frac{\rho_{b}}{\rho_{w}}$ is the ratio of the density of the sea bottom material *ρ_b_* to the water density *ρ_w_* near the sea bottom, and is the incident angle on the sea bottom boundary.

The reflections on the medium boundaries and the refractions in the heterogeneous ocean medium make the final received signal a mixture of multiple attenuated, delayed and phase-shifted input signals. Therefore, collecting information (including the number, the amplitudes and the phases) on rays that emit from the source node towards the destination node is critical in determining the received signals. We will incorporate these influences in SAM.

### 2.2. Motivation

The underwater environment varies greatly depending on the geographic location. Underwater networks may be deployed in areas of shallow water (tens of meters to hundreds of meters), or in areas of great depths (thousands of meters). The nodes may be deployed near the surface, suspended in the middle, or anchored to the seabed. Climates, which can greatly affect the propagation of waves, are drastically different depending on where the network resides [25]. Existing channel models are either too complex and scales poorly, or are inaccurate as they use empirical formulas for transmission loss prediction, making approximations that neglect the boundary (at the sea surface or the sea floor) reflection losses, the multi-path phenomenon, and the ray bending in the diverse ocean environments. We aim to bridge the gap of existing work by introducing a new underwater channel model and evaluating its impacts on network design. Our approach is based on the geometrical tracing of sound rays in specific ocean environments, using the minimum necessary oceanographic theory while retaining the fundamental features which are critical for network designers.

## 3. Stratified Acoustic Model Development

Our goal is to provide an efficient simulation-friendly underwater channel model to accurately predict the transmission loss through capturing the characteristics of the specific deployment site. As the ocean environment varies greatly from site to site, it is hard to find a universal closed-form expression applicable to model the propagation of sound in various ocean media. In this paper, we aim to propose a stratified acoustic model based on the geometrical ray tracing technology, incorporating the details of the target environment, to predict the transmission loss. Our proposed stratified acoustic model (SAM) is developed in three steps:

(1) Initial geometrical ray tracing using a discrete set of rays to map out the sound field.
(2) Determining the eigen-rays, which are defined as rays connecting the source node and the destination node, and calculating the amplitude and phase for each eigen-ray taking the bounces on the boundaries into consideration.
(3) Computing the transmission loss.

### 3.1. Initial Geometrical Ray Tracing

In SAM, the source node is assumed to be an omni-directional transmitter. The characteristics of the bathymetry, the sound speed profile, the source location and the destination location, are required as input to SAM.

Before presenting the details of the ray tracing, we present the categories of the eigen-rays and define common terminologies that will be used throughout the paper. Considering the example shown in Figure 2, *z_S_* and *z_D_* are the depths of the source and the destination, respectively, where *z_S_* < *z_D_* (due to space limitations, only the case *z_S_* < *z_D_* is analyzed in this paper, however, for *z_S_* ≥ *z_D_*, relevant analysis can be similarly carried out). According to the form and the order of the reflection, the eigen-rays can be grouped into five types: (1) direct rays propagating via a refracted path only, denoted *Direct*; (2) rays that make the first and last boundary reflections both on the sea-surface before arriving at the destination, denoted by *SS*; (3) rays that make the first boundary reflection on the sea-surface and the last boundary reflection on the sea bottom before arriving at the destination, denoted by *SB*; (4) rays that make the first boundary reflection on the sea-bottom and the last boundary reflection on the sea-surface before arriving at the destination, denoted by *BS*; (5) rays that make the first and the last boundary reflections both on the sea-bottom before arriving at the destination, denoted by *BB*.

**Figure 3.**
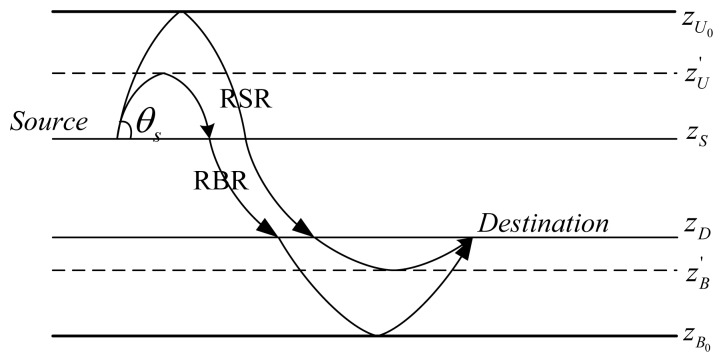
Refracted surface-reflection ray (RSR) and refracted bottom-reflection ray (RBR).

According to Snell's law in Equation (1), sound bends locally towards regions of lower sound speed. In an extreme case, when there is a sound speed *c_cri_* in the water column at depth *z_cri_* satisfying *c_cri_* = *c_s_*/cos *θ_S_*, the ray with a take-off angle *θ_S_* emitting from the source node at depth *z_S_* with the sound speed of *c_S_*, will turn before hitting the sea boundaries (sea surface/bottom) as shown in Figure 3.

Specifically, if *z_cri_* > *z_S_*, the ray will bounce off the sea surface and turn towards the destination before hitting the sea bottom (Refracted Surface-reflection Ray, RSR, shown in Figure 3); if *z_cri_* < *z_S_*, the ray will turn before hitting the sea surface, and then bounce at the sea bottom before reaching the destination (Refracted Bottom-reflection Ray, RBR).

Therefore, besides SS, BB, SB, and BS shown in Figure 2, these two kinds of additional rays, RSRs and RBSs, shown in Figure 3, should also be dealt with (a ray may go towards the sea surface, but turns before hitting the surface, and then turns again towards the destination before hitting the sea bottom, but this can be considered as a “Direct” ray). Fortunately, for RSRs, we can imagine that there is a virtual bottom boundary at the depth $z_{B}^{\prime }=z_{\text{cri}}$ (when *z_cri_* > *z_S_*), and rays bounce between the virtual bottom boundary and the sea surface alternately; for RBRs, it can be understood as rays bouncing off between a virtual sea surface boundary at the depth $z_{U}^{\prime }=z_{\text{cri}}$ (when *z_cri_* < *z_S_*) and the sea bottom.

In this case, RSRs and RBRs can be considered as special cases of SS, BB, SB, BS. To incorporate RSRs and RBRs in SS, BB, SB, BS, for a ray emitted from the source depth *z_S_* (the sound speed at depth *z_S_* is denoted by *c_S_*) with a take-off angle *θ_S_*, we firstly compute the critical sound speed *c_cri_* = *c_s_*/cos *θ_S_*, and determine whether there exists a depth *z_cri_* with the sound speed being *c_cri_*. Then, we define the actual sea surface depth *z_U_*, the actual real sea bottom depth *z_B_*, the actual surface coefficient *c_s_* (for the ray with a take-off angle *θ_S_* emitting from the source node at depth *z_S_* with the sound speed of *c_S_*, if the sound speed at *z_cri_* depth is *c_cri_* satisfying *c_cri_* = *c_s_*/cos *θ_S_*, according to Snell's law, the sound ray will be totally reflected at *z_cri_*. In this case, it is assumed that there is a virtual sea surface, and a phase shift of 180° is caused by the interaction with the virtual sea surface. Therefore, the interaction coefficient on this virtual sea surface is −1) and the actual bottom reflection coefficient *ϒ_b_* as follows:

(5) $$z_{U}=\{\begin{array}{l} z_{\text{cri}},\;\text{if}\;z_{\text{cri}}<z_{S}\\ 0,\;\text{otherwise} \end{array}$$

(6) $$z_{B}=\{\begin{array}{l} z_{\text{cri}},\;\text{if}\;z_{\text{cri}}>z_{S}\\ z_{\text{MAX}},\;\text{otherwise} \end{array}$$

(7) $${ϒ}_{s}=\{\begin{array}{l} -1,\;\text{if}\;z_{\text{cri}}<z_{S}\\ \gamma_{s},\;\text{otherwise} \end{array}$$

(8) $${ϒ}_{b}=\{\begin{array}{l} -1,\;\text{if}\;z_{\text{cri}}>z_{S}\\ \gamma_{b},\;\text{otherwise} \end{array}$$

The coefficients *γ_s_* and *γ_b_* are defined as in Equations (3) and (4), respectively. Therefore, RSRs and RBRs in Figure 3 can be incorporated into the classification of SS, BB, SB, BS, where the letter S indicates bouncing at the upper boundary, either the real sea surface or the imaginary sea surface, and the letter B indicates bouncing at the lower boundary, either the real sea bottom or the imaginary sea bottom. Note that if *z_S_* < *z_cri_* < *z_D_*, the ray with the take-off angle *θ_S_* will be discarded because it will turn before hitting the depth of the destination so that it has no possibility to arrive at the destination node.

Further, to incorporate more eigen-rays with the above classification, as in References [20,27], we extend these notations with a subscript *n*, *i.e.*, *SS_n_*, *SB_n_*, *BS_n_*, *BB_n_*, to define a ray with a bounce time order *n*. The subscript *n* denotes the ray's interaction times on the specific boundary which is identified by the second letter in the notation, e.g., *SS_n_* means the ray bounces *n* times totally on the sea surface before arriving at the destination. Then, with the assumption that the bounces between the sea surface and the sea bottom are alternative, it is easy to infer that there are *n*−1 bounces on the sea bottom for *SS_n_*. For instance, the paths shown in Figure 2 are with *n* = 1.

The initial ray tracing is done by launching a large number of rays (e.g., in the examples shown in Section 4, 18,000 rays are used) with angles selected to cover the entire space between the source and the destination. For a ray that starts from the source with a specific grazing angle *θ_S_*, the horizontal distance (*V*) covered by this ray with a grazing angle *θ_S_* when it hits the depth of the destination, is defined as

(9) $$\text{V}=\int_{z_{S}}^{z_{D}}\frac{dz}{\tan\theta (z)}$$

where z_S_ and z_D_ are the depths of the source and the destination, respectively, and θ(*z*) is the grazing angle of the ray at the depth of *z*. Moreover, the horizontal distance between the source and the destination is denoted by *r_SD_*. To decide whether it is an eigen-ray, the assumption confirmation method is applied in our study, that is, we check the difference between *V* and *r_SD_*. If

(10) $$\text{V}-r_{SD}=0$$

the ray with a take-off angle θ*_zS_* is taken as an eigen-ray.

### 3.2. Computation of Signal Losses for Each Path

To obtain the final form of the received signals at the destination node, information on the amplitude (which depends upon the spreading losses, absorption losses, and bounce losses at the boundaries), and the phase (which consists of the phase shift introduced by delay, and the phase shift caused by the complex reflection coefficients at the boundaries) for each ray should be collected.

**Figure 4.**
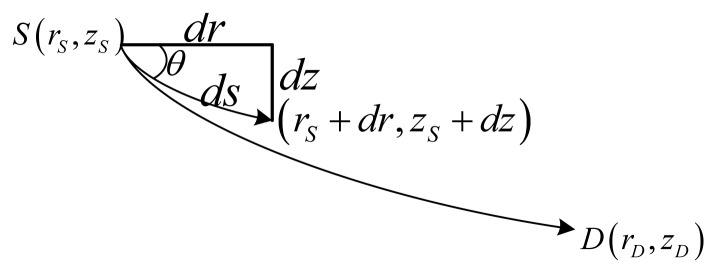
A micro unit of ray tracing.

Firstly, the direct ray is studied. As shown in Figure 4, the curvilinear length *l_Direct_* and the delay *t_Direct_* for a ray from *S*(*r_S_*, *z_S_*) to *D*(*r_D_*, *z_D_*) can be written as Equations (11) and (12), respectively, where *c_S_* is the sound speed at the source's depth, and *θ_S_* is the horizontal incident angle at the source's depth:

(11) $$l_{\text{Direct}}=\int_{z_{S}}^{z_{D}}\frac{dz}{\sin\theta (z)}$$

(12) $$t_{\text{Direct}}=\int_{z_{S}}^{z_{D}}\frac{n^{2}(z)dz}{c_{S}\sqrt{n^{2}(z)\cos^{2}\theta_{S}}}$$

**Figure 5.**
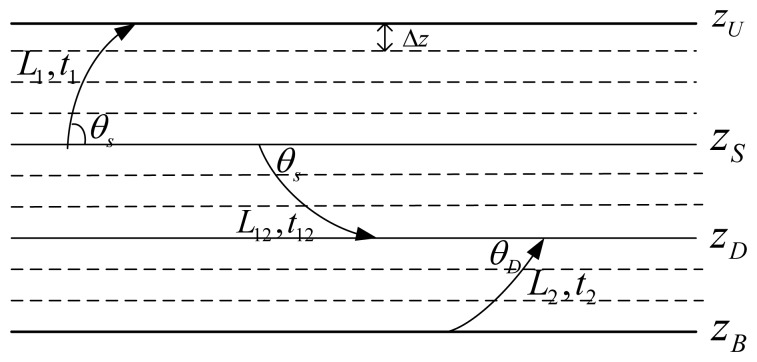
Specific forms of pathlets.

Secondly, the actual lengths of each kind of propagation path shown in Figure 2, denoted *l_SSn_*, *l_SBn_*, *l_BSn_* and *l_BBn_*, respectively, can be expressed as a combination of three pathlets shown in Figure 5, where $L_{1}=\int_{z_{S}}^{z_{U}}\frac{dz}{\sin\theta (z)}$ is the length of the ray with a horizontal incident angle *θ_S_* from the depth of *z_S_* to the sea surface *z_U_*, $L_{12}=\int_{z_{S}}^{z_{D}}\frac{dz}{\sin\theta (z)}$ is the length of the ray with a horizontal incident angle *θ_S_* starting at the depth of *z_S_* and arriving at the depth of *z_D_*, and $L_{2}=\int_{z_{B}}^{z_{D}}\frac{dz}{\sin\theta (z)}$ is the length of the ray starting from the sea bottom *z_B_* and arriving at the depth of *z_D_* with a horizontal incident angle *θ_D_*. Therefore, *l_SSn_*, *l_SBn_*, *l_BSn_*, and *l_BBn_*, by:

(13) $$\{\begin{array}{c} l_{SS_{n}}=2\text{nt}L_{1}+(2n-1)L_{12}+2(n-1)L_{2}\\ l_{SB_{n}}=2nL_{1}+(2n-1)L_{12}+2nL_{2}\\ l_{BS_{n}}=2nL_{1}+(2n+1)L_{12}+2nL_{2}\\ l_{BB_{n}}=2(n-1)L_{1}+(2n-1)L_{12}+2nL_{2} \end{array}$$

Furthermore, since the received signal at the destination node is a superposition of ray arrivals with different amplitudes and phases, there is also a need to obtain the delay for each arrival. Similar to the calculation of lengths of these rays, with the delays of the three types of pathlets shown in Figure 5, denoted *t*_1_, *t*_12_, and *t*_2_, respectively, the delays of different propagation rays, denoted *t_SSn_*, *t_SBn_*, *t_BSn_*, and *t_BBn_*, can be written as:

(14) $$\{\begin{array}{c} t_{SS_{n}}=2nt_{1}+(2n-1)t_{12}+2(n-1)t_{2}\\ t_{SB_{n}}=2nt_{1}+(2n-1)t_{12}+2nt_{2}\\ t_{BS_{n}}=2nt_{1}+(2n+1)t_{12}+2nt_{2}\\ t_{BB_{n}}=2(n-1)t_{1}+(2n-1)t_{12}+2nt_{2} \end{array}$$

For the received signal, bounce losses at the boundaries are exponential functions of bounce times. Therefore, based on Equations (2) and (4), according to the bounce times shown by the notations, the combined loss of sound pressure due to repeated surface and/or bottom reflections for each type of the sound ray can be further given by:

(15) $$\{\begin{array}{c} R_{SS_{n}}={ϒ}_{s}^{n}{ϒ}_{b}^{n-1}\\ R_{SB_{n}}={ϒ}_{s}^{n}{ϒ}_{b}^{n}\\ R_{BS_{n}}={ϒ}_{s}^{n}{ϒ}_{b}^{n}\\ R_{BB_{n}}={ϒ}_{s}^{n-1}{ϒ}_{b}^{n} \end{array}$$

Where *n* = 1,2,…

Another factor that decreases the sound pressure during propagation in water is the absorption by the medium. The absorption coefficient α(*f*) is defined in Reference [25], which can be expressed empirically using Thorp's formula in decibels per kilometer for *f* in kilohertz as:

(16) $$10\mathrm{lg}\alpha (f)=0.11\frac{f^{2}}{1+f^{2}}+44\frac{f^{2}}{4100+f^{2}}+2.75\times 10^{-4}f^{2}+0.003$$

Since the path length (described in Equation (13)) of the actual trajectory of the sound ray is inversely proportional to the amplitude of the received signal (this influence is also called the spreading loss), the bounce losses are described in Equation (15) and the absorption loss (*dB/km*) within the water column is as in Equation (16), the amplitudes for each type of signals can be written as:

(17) $$\{\begin{array}{c} A_{\text{Direct}}=\frac{1}{l_{\text{Direct}}\alpha {\left(f\right)}^{\frac{l_{\text{Direct}}}{2\times 10^{-3}}}}\\ A_{SS_{n}}=\frac{R_{SS_{n}}}{l_{SS_{n}}\alpha {\left(f\right)}^{\frac{l_{SS_{n}}}{2\times 10^{-3}}}}\\ A_{SB_{n}}=\frac{R_{SB_{n}}}{l_{SB_{n}}\alpha {\left(f\right)}^{\frac{l_{SB_{n}}}{2\times 10^{-3}}}}\\ A_{BS_{n}}=\frac{R_{BS_{n}}}{l_{BS_{n}}\alpha {\left(f\right)}^{\frac{l_{BS_{n}}}{2\times 10^{-3}}}}\\ A_{BB_{n}}=\frac{R_{BB_{n}}}{l_{BB_{n}}\alpha {\left(f\right)}^{\frac{l_{BB_{n}}}{2\times 10^{-3}}}} \end{array}$$

Where *n* = 1,2,…

### 3.3. Transmission Loss Calculation

With the abovementioned initial geometrical ray tracing and computation of signal losses for each path, the spreading losses determined by calculating the overall path length of the actual trajectory of sound ray (expressed in Equation (13)), the absorption losses in *dB/km* computed in Equation (16), the bounce influences including amplitudes and phase shifts described in Equation (15), and the phase shifts introduced by the delay computed in Equation (14), the channel impulse response *h*(*t*) can be expressed as [34]:

(18) $$\text{h}(t)=A_{\text{Direct}}\delta (t-t_{\text{Direct}})+\sum_{n=1}^{\infty }\left(A_{SS_{n}}\delta (t-t_{SS_{n}})+A_{SB_{n}}\delta (t-t_{SB_{n}})+A_{BS_{n}}\delta (t-t_{BS_{n}})+A_{BB_{n}}\delta (t-t_{BB_{n}})\right)$$

Specifically, if the ocean medium is regarded as a communication channel, the received signal *y*(*t*) at the destination is the convolution of an input *x*(*t*) with the relevant channel impulse response *h*(*t*). The transmission loss, denoted by *TL*, in dB, can be calculated as:

(19) $$\text{TL}=10\mathrm{lg}\frac{E_{s}\left(x(t)\right)}{E_{s}\left(y(t)\right)}$$

where $E_{s}(\cdot )=\int_{t}{\left|\cdot \right|}^{2}dt$ is the function for computing the signal energy in the time domain. Furthermore, according to Parseval's Equation, the energies for the input signal *x*(*t*) and the received signal *y*(*t*) in the time domain are equal to that in the frequency domain [35], and the transmission loss in Equation (19) can be further computed by:

(20) $$\begin{array}{ll} \text{TL} & =10\mathrm{lg}\frac{E_{f}\;(x(t))}{E_{f}\;(y(t))}\\ & \overset{\left(a\right)}{=}10\mathrm{lg}\frac{E_{f}\;(x(t))}{E_{f}\;(x(t)⊗h(t))}\\ & =10\mathrm{lg}\frac{\int_{-\infty }^{\infty }{\left|\text{FFT}(x(t))\right|}^{2}df}{\int_{-\infty }^{\infty }{\left|\text{FFT}(x(t)⊗h(t))\right|}^{2}df}\\ & \overset{\left(b\right)}{=}10\mathrm{lg}\frac{1}{\int_{-\infty }^{\infty }{\left|\text{FFT}(h(t))\right|}^{2}df}\\ & =-10\mathrm{lg}(E_{f}\;(h(t))) \end{array}$$

where $E_{f}\;(\cdot )=\int_{-\infty }^{\infty }{\left|\text{FFT}(\cdot )\right|}^{2}df$ is the function for computing the signal energy in the frequency domain, (a) is achieved by y(t) = x(t)⊗h(t) and ⊗ is the convolution operation, *FFT(h(t))* is the Fast Fourier Transform (FFT) of *h(t)*, and (b) is achieved by taking the input signal *x(t)* as an impulse signal.

However, not all the eigen-rays should be considered to compute the transmission loss. There are two methods to determine how many rays should be incorporated. One is to limit the number of terms to include only those with significant amplitudes (for example, only rays with amplitudes larger than 1% of the strongest ray). Another is to consider the delay difference between a candidate ray and the first arrival ray. In digital communications, information is transmitted by means of symbols, each with duration *T_s_*. It is postulated that at the destination the signal is analyzed within one symbol duration *T_s_*. Therefore, to estimate the signal strength, only rays with delay differences less than *T_s_* are considered, while multipath signals with delay differences larger than *T_s_* will be considered as the interference signals to the subsequent transmitted signals. In this paper, we adopt the second method to determine the sound rays for transmission loss calculation.

### 3.4. Implementation of SAM

**Figure 6.**
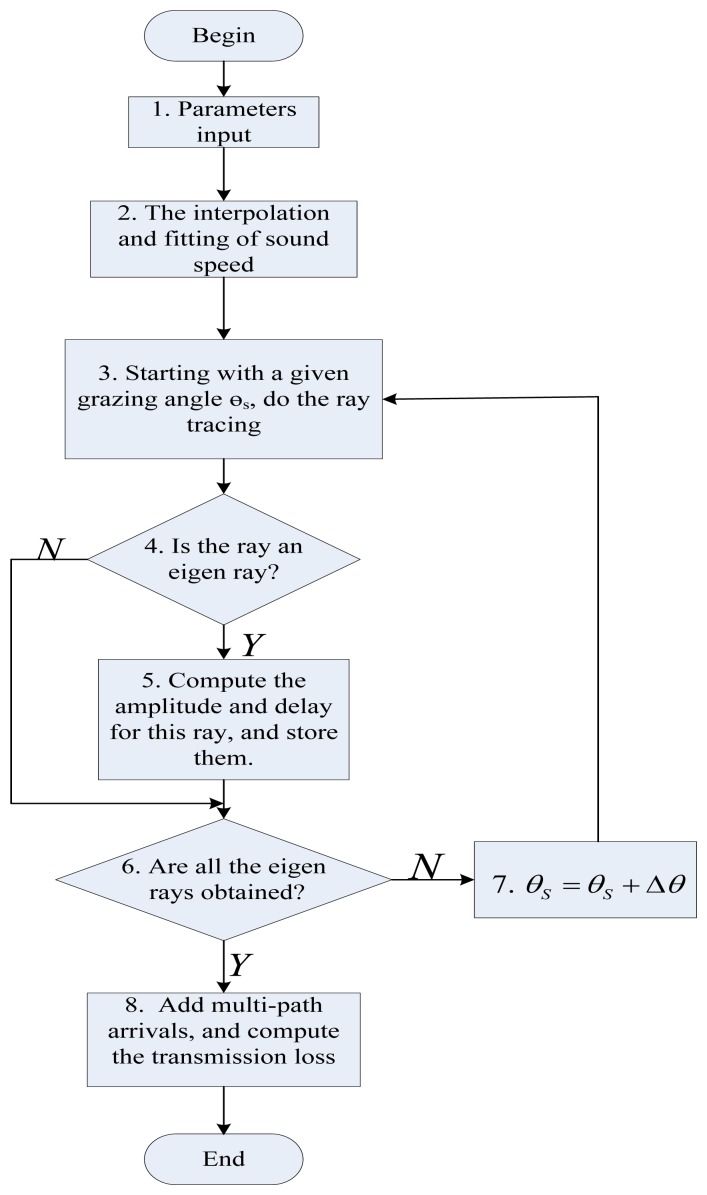
Flowchart of the implementation of SAM.

Currently the algorithm for computing the transmission loss between a given pair of underwater nodes as shown in Figure 6 is achieved in MATLAB (as mentioned in Section 1, our goal in this paper is to develop an underwater channel which is accurate and applicable for large-scale underwater acoustic sensor network. Considering that Matlab may not be suitable tool for large-scale networks, we will implement SAM in the popular network simulators such as OPNET and NS-2 in the future). The environmental information should be known in advance, including the sound speed profile, the depth of the sea, the locations of the source and the destination, the sea state (e.g., the average wind speed at the sea surface) and the sea floor characteristics (e.g., the density of the sea bottom sediment, the sound speed in the sea bottom sediment, and the bathymetry of the sea floor, e.g., flat or skewed) as specified in Step 1 of Figure 6. The output is the transmission loss of the underwater channel.

**Figure 7.**
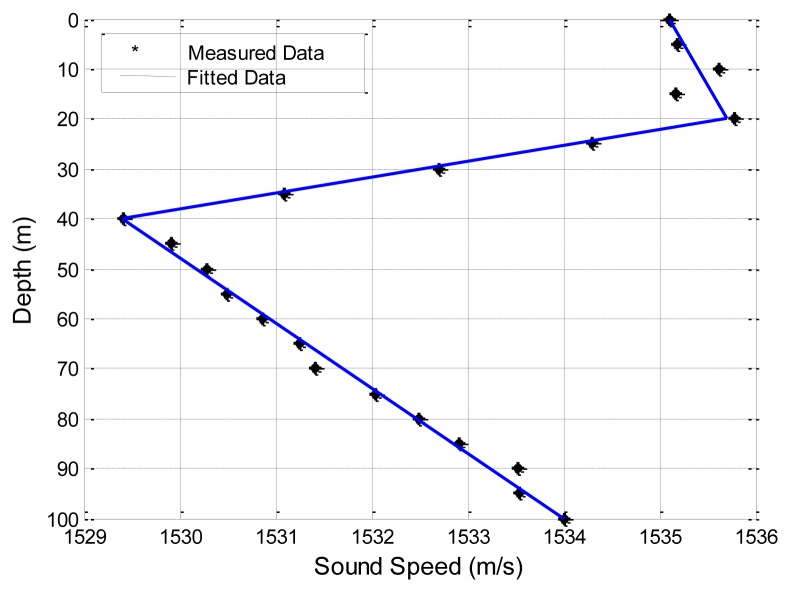
Sound speed variation with depths in a zone in the China sea.

Often, the ocean sound speed profile (SSP) is provided at discrete depths and careful thoughts must be given to properly interpolate this data in Step 2. Here we use a piecewise linear fitting method to approximate the variation of sound speed with depths. Take an example shown in Figure 7, the dots are measured sound speeds at discrete depths in a zone of the China Sea. The sound speed increases slightly with depths for 0–20 m, decreases with depths for 20–40 m, and increases with depths for 40–100 m. Therefore, we approximate this sea volume with three layers as plotted in three straight lines in Figure 7.

Then, we select the angle interval [−90°, 90°] to specify the sector that will be used during the ray tracing process, and launch rays with incremental angle of Δθ. It is assumed that the angles are specified in declination, *i.e.*, zero degree corresponds to a horizontally launched ray, and a positive angle corresponds to a ray launched towards the bottom. Here, to reduce the probability of missing the important eigen-rays, the step length Δθ in Step 7 is set to a sufficiently small but computationally feasible value of 0.01°.

Based on Equation (9), the horizontal distance *V* traversed by this ray can be obtained by tracing the trajectory of each ray referred to in Step 3. Then, to decide whether a ray that starts from the source with a specific grazing angle *θ_s_* is an eigen-ray or not in Step 4, the assumption confirmation method is applied, that is, if Equation (10) is satisfied, this propagation path with a take-off angle *θ_s_* is taken as an eigen-ray. Then, based on Equation (14), if the delay is less than one symbol duration *T_s_* (e.g., 1 ms; *T_s_* is the inverse of the achievable data rate. Here it is assumed the data rate is 1 kbps, which can be achieved by some off-the-shelf underwater modems (e.g., UWM2000H made by LinkQuest Inc., San Diego, CA, USA[36])) this ray with a take-off angle *θ_s_* is one of the rays that will be counted when calculating the underwater channel response in Equation (18). Then, based on Equations (13) and (14), computation in Step 5 can be completed. After tracing all the rays, finally, according to Equation (20), the transmission loss is output in Step 8.

## 4. Model Verification and Summary

### 4.1. Model Verification

The accuracy of a new model can be verified by comparisons with results using previously validated models and with experimental data. In our work, results from standard methods (Ray Model, Normal Mode, Wavenumber Integration, and Parabolic Equation (PE)) are used. They are chosen for comparison because they are the most popular and accurate underwater acoustics models [12,14]. Since there are many implementations for each method, four well-known models (details can be found in Reference [37]), namely, Bellhop for ray theory, KRAKEN for normal mode, SCOOTER for wavenumber integration, and RAM for PE, are incorporated in this paper. Besides, one widely used approximation method for predicting transmission loss in UANs, having an expression of 15lgd + d × 10^−2^*lgα*(*f*) (*d* is the Euclidean distance between the source and the destination), denoted Empirical, is also included. Two sets of experimental data are used to evaluate the performance of SAM.

**Figure 8.**
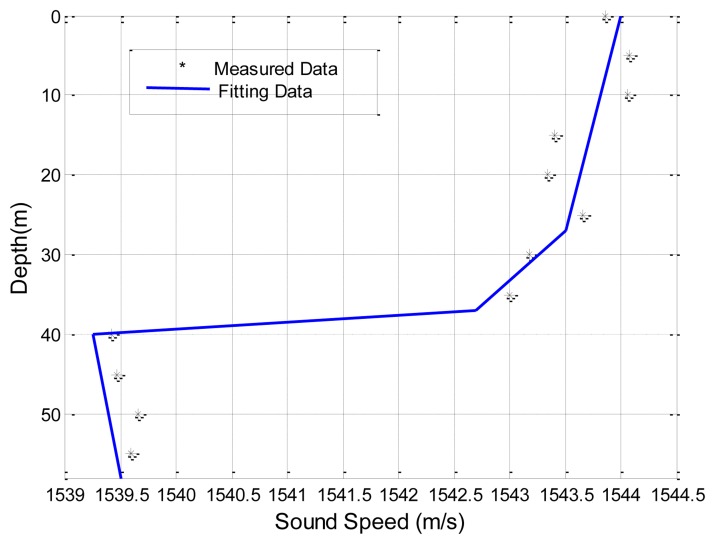
Sound speed variations with depths in an Australian ocean site.

**Figure 9.**
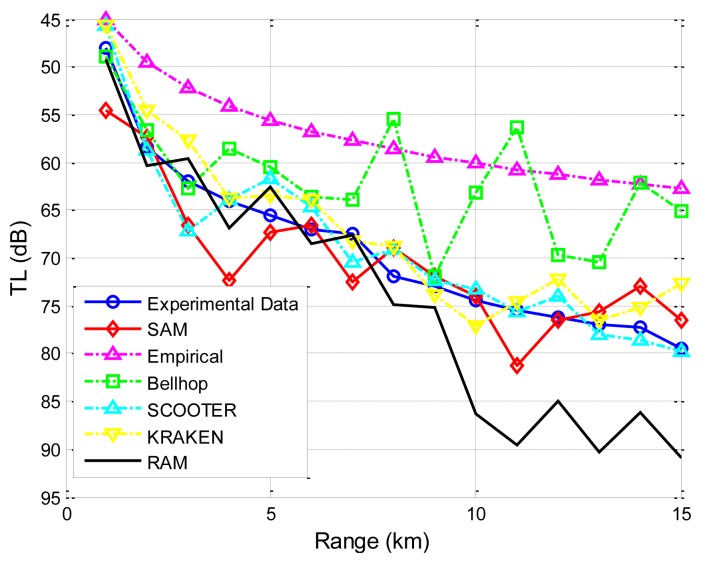
Transmission loss *vs.* range under different models in an Australian ocean site.

The first set of experimental data presented is obtained by the Maritime Operations Division of Defence Science and Technology Organization, Australia [38]. Both the signal source and destination are deployed at a depth of 18 m. The sea has a nearly uniform depth of 58 m. The sound speed profile (SSP) is shown in Figure 8. A seafloor database which includes information on the density of the sea bottom sediment, the sound speed in the sea bottom sediment, and the bathymetry of the sea floor, e.g., flat or skewed, is used Reference [38]. Specifically, the sediment is sand-silt-clay, with a density of 1,660 *kg/m^3^* and the sound speed is 1,570 *m/s*. The sea water density near the sea bottom is 1,030 *kg/m^3^*, and the air media above the sea surface is approximated as a vacuum. Transmission losses predicted using SAM, Bellhop, KRAKEN, SCOOTER and RAM, are shown in Figure 9. All predictions are made with the same carrier frequency of 250 Hz.

The plots in Figure 9 indicate that our proposed model and the four physics-based models follow a similar trend and show better agreement with measured transmission loss values than the empirical approximation does. Since the empirical approximation does not account for multi-path effects, this trend is expected. It is noted that, for RAM and Bellhop, as the communication range increases, the differences between predicted results and measured experimental results become greater. This is because in long-range underwater acoustic communications, severe multi-path effects will occur, and the received signal is the sum of a large number of these multipath arrivals, each of which is modeled as a complex stochastic process. For effective channel modeling, the variation of each path signal caused by the sea boundary (the sea surface and the sea bottom) should be taken into account. However, for RAM, the scattering on the rough surface/bottom boundary is not dealt with Reference [39]; while Bellhop does not implement the rays' phase shifts, which would be necessary to provide a better treatment of the reflection coefficient [4,12]. However, the variance between experimental data and predicated results by KRAKEN, SCOOTER and SAM is mostly less than 5 dB. Both KRAKEN and SCOOTER calculate an equivalent reflection coefficient for the sea surface/bottom and incorporate it into the model in a tabular form with essentially perfect accuracy, and the performance degradation is due to the coarse sampling of the reflection coefficient. Moreover, SAM utilizes the popular surface/bottom reflection model that has closed-form expression to incorporate the boundary influences on the rays' amplitudes and the phases. It is noted that there are still deficiencies in the four physics-based models and in SAM. For the physics-based models, the accuracy depends on the environmental parameters, boundary conditions, and the approximation adopted. Therefore, lack of accurate real-time environmental input and approximation errors might explain the discrepancies [4,12,25]. For SAM, inaccuracies may be due to inaccurate environmental inputs, insufficient resolution in discretizing the ocean column into multiple sub-layers, insufficient number of rays traced to generate the eigen-rays, and the sea surface/bottom reflection models chosen.

**Figure 10.**
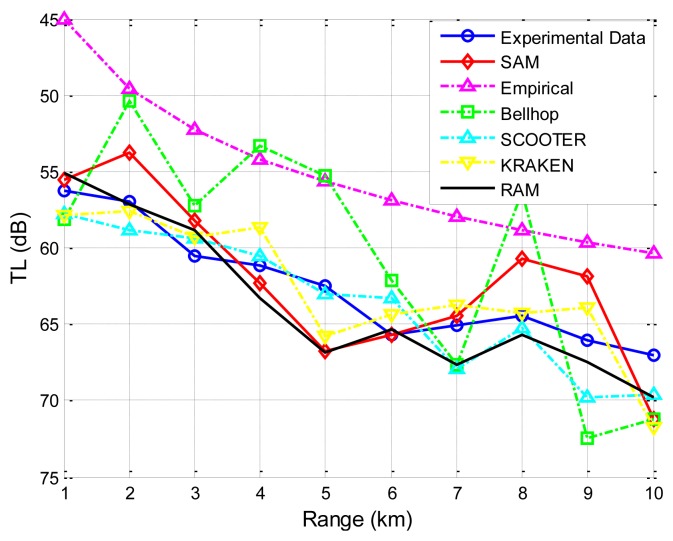
Transmission loss *vs.* range under different models in China sea.

The second experimental data set is collected in a field experiment by the Acoustics Institute of the Chinese Academy of Sciences [30]. The sea floor is smooth in the experimental zone for a range of 10 km, and the average sea depth is 100 m. The sound speed profile is given in Figure 7. Wind speed is 6–8 m/s. Average wave height at the sea surface is about 0.85 m. Moreover, the ratio of the densities of the sea floor to the sea water is 1.95, and the ratio of the sound speed in the sea to that at the sea floor is 0.86. Thus, the sound speed in the sea bottom is 1,786.05 m/s (1,536/0.86 = 1,786.05). The source and the destination are located 6 m and 5 m underwater, respectively. The carrier frequency is 600 Hz. As shown in Figure 10, it is obvious that SAM and the four physics-based models have better prediction of the transmission loss than the empirical approximation.

Furthermore, taking the second experiment shown in Figure 10 as an example, we studied the influences of the number of water layers (*i.e.*, the sea depth divided by Δz) and the number of rays traced (*i.e.*, 180° divided by Δθ) on both the accuracy and the computational complexity. The results are shown in Table 1. Specifically, the accuracy is evaluated by the differences between the experimental results and the predicted results by SAM (denoted Error in Table 1), and the computational complexity is evaluated by the computation time (denoted Time in Table 1) on a PC with AMD Athlon 64 × 2, CPU 5,000+, 2.60 GHz, and 2 GB processor.

### 4.2. Model Summary

Underwater propagation models may be categorized as follows. One category is physics-based models, such as ray theory, normal mode, wavenumber integration and the parabolic equation, and they are applicable for different frequencies. Specifically, ray theory models calculate the transmission loss on the basis of ray tracing and throw away the phases of the individual rays [12]. Moreover, except for the ray theory models, the computational complexity of other models is frequency-dependent. Therefore, computationally intense processing is required, rendering them impractical for large scale underwater acoustic network simulations. The other category is the fitting-based empirical approach, and such simplified models have been widely adopted in simulations and incorporated into the software packages NS2 and OPNET. They simply sum up the spreading loss, the absorption loss, the surface loss and the bottom loss to get an estimation of the transmission loss. Actually, the surface loss and the bottom loss are not always included, and when they are included, they are simply added up, and the phase shifts are ignored. In SAM, we utilize geometrical ray tracing and make use of the small scale fading (here, the small scale fading is the rapid fluctuations of the amplitude and phase of a micro-path acoustic signal when interacted with the sea medium's boundaries (either the sea surface or the sea bottom) over a short period of time) to predict the large-scale transmission loss. Specifically, the phase shifts experienced by the rays are incorporated in SAM.

**Figure 11.**
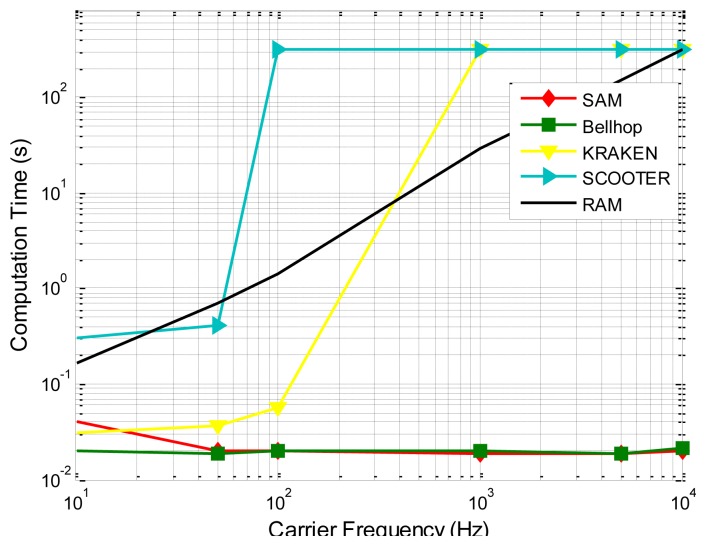
Computational time *vs.* carrier frequency.

Since the phase shifts corresponding to multiple paths may cause constructive or destructive interference on the received signal, they play an important role in the transmission loss prediction, as is demonstrated by SAM's better prediction ability than Bellhop as shown in Figures 10 and 11 (since it is hard for a single channel model to precisely capture all the characteristics of the underwater channel, the better predictive ability provided by SAM in these two experiments may not necessarily mean that SAM will always give the best prediction). Besides, the surface/bottom boundary influence is incorporated based on the current standard model with closed-form expressions. More importantly, the average computational times under different models for a single transmission on a PC with AMD Athlon 64 × 2, CPU 5,000+, 2.60 GHz, and 2 GB processor, are compared in Figure 11, showing that the computational complexity of SAM and Bellhop is independent of the frequency while the computational complexity of KRAKEN, SCOOTER, and RAM increases quickly with the carrier frequency [37]. SAM enjoys, for some runs, three to four orders of magnitude lower computational complexity. Table 2 shows a comparison between the Empirical model, SAM, Bellhop, KRAKEN, SCOOTER, and RAM.

## 5. Application of SAM

**Figure 12.**
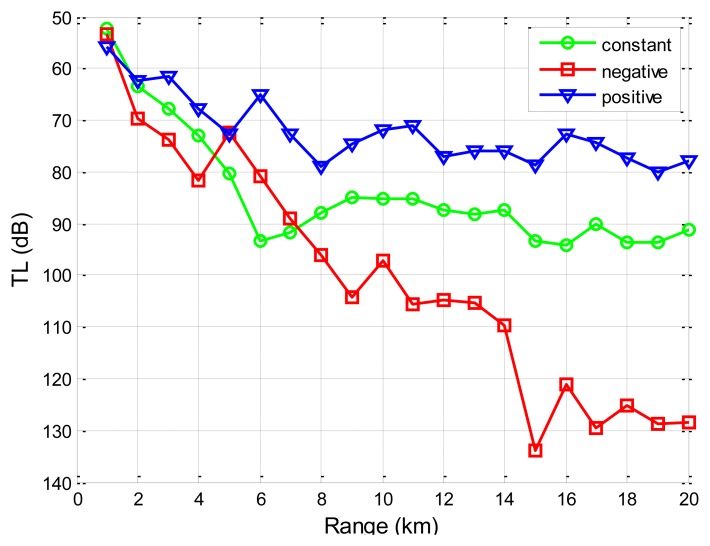
Transmission loss *vs.* horizontal range, under different sound speed profiles.

Firstly, we use SAM to show the influences of the stratified ocean medium on the transmission loss for the underwater channel. The source node is located 50 m underwater, and the destination node is located 20 m underwater. The sound speed for the Taiwan strait, as reported in Reference [40], increases linearly with a positive gradient with depths in January (1,510–1,515 m/s), stays constant across the water volume in May (1,527 m/s), and decreases linearly with depths in August (1,540–1,530 m/s) of 1998 (water depth 60 m). As shown in Figure 12, we find that the distribution of sound speed in the water has an impact on the propagation of the sound.

It is because different sound speeds result in different ray paths during the propagation of sound in the water based on Equation (16), rendering the received acoustic signals different. Since the empirical formula does not consider the distribution of the sound speed and has a uniform expression for various environments, they cannot predict transmission loss differences under different sound speed profiles.

**Figure 13.**
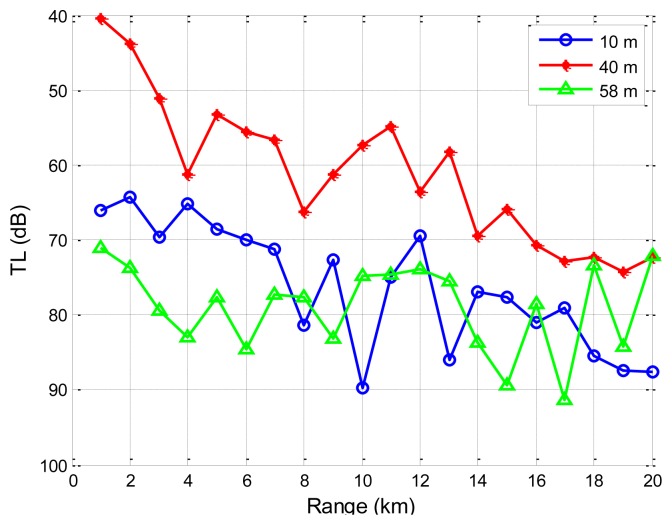
Transmission loss *vs.* range, under different receiver depths.

Secondly, we address the impacts of source depths on the transmission loss through SAM. The sound speed profile and oceanographic parameters from Reference [38] are used. Details can be found in Section 4.1. The source node is located 40 m underwater. The carrier frequency is 1,000 Hz. The transmission loss performance using SAM is shown in Figure 13 for three different destination depths: 10, 40, and 58 m underwater. Noting that the y axis is upside down, the transmission loss is the smallest when the destination is located 40 m underwater. From Figure 9, we can find that the sound speed is minimal at 40 m deep. According to Snell's Law, an acoustic ray always bends towards the side with smaller sound speed. Therefore, eigen-rays connecting the source and the destination, both located 40 m underwater, propagate via refracted paths without encountering reflection losses at the sea surface or the seafloor, giving the smallest transmission loss. However, for destinations located at 10 and 58 m, near the sea surface and the sea floor, the rays from the source to the destination interact with the sea surface and sea floor, resulting in additional energy loss. These results indicate that the underwater acoustic communication performance variation is largely aligned with source/destination configurations.

Besides, from Figures 12 and 13, it is seen that transmission loss for underwater acoustic communication is not monotonically increasing or decreasing. There are regions where the transmission loss spikes or drops suddenly. These fluctuations represent regions where multi-path reflections are combined constructively or destructively. However, the empirical approximation model does not reflect these regions and provides a less realistic smoothed approximation of the transmission loss. It is noted that these regions where transmission loss spikes or drops suddenly should be given special attention when one performs topology optimizations and protocol designs for UANs.

## 6. Conclusions

In this paper we propose an efficient simulation-friendly and accurate model to predict the transmission loss by capturing the characteristics of a specific underwater deployment site. The computational complexity of our model is mainly due to geometrical ray tracing and is frequency-independent, rendering it applicable for large scale underwater acoustic network simulations. Comparisons in two ocean regions have been conducted to verify the proposed model, and satisfactory agreement is demonstrated with measurement results and with other highly computationally intensive physics-based models. Our proposed model is also superior to empirical models, which do not account for the variations of ocean regions, leading to inaccurate predictions of propagation loss. Therefore, our model bridges the gap between the overly simplified empirical model and the computationally intensive physics-based models. In the future, we will study how the channel models impact the protocol designs for underwater acoustic sensor networks.

## Figures and Tables

**Table 1.** Impact of parameters on both accuracy and computation time.

**No. of Water Layers**	**Error (dB)**	**Time (s)**	**No. of Rays Traced**	**Error (dB)**	**Time (s)**
1	10.9697	4.1633	180	13.2584	1.594515
2	8.5834	4.883395	1,800	6.8564	3.971890
3	4.1671	5.626749	18,000	4.1671	5.626749
4	3.9388	5.723302	36,000	4.1659	13.137566
5	2.9026	8.895388	72,000	4.1633	21.429046

**Table 2.** Comparisons of different computation models for transmission loss.

	**Characteristics**	**Accuracy**	**Computation Complexity**
**Empirical Calculation** **(Thorp)**	Simple Straight line propagation assumed Multi-path propagation not included	Poor (Error > 10 dB)	Low (*μs*)
**Ray theory** **(Bellhop)**	Efficiency and accuracy in tracking rays Throw out the phases of each ray	Good For ideal sea conditions For short ranges (Error < 10 dB)	Medium (a few seconds)
**Normal Mode** **(KRAKEN)**	Integral representation of the wave equation The number of modes required increases in proportion to the frequency	Very good below 500 Hz (Error < 2dB)	High (thousands of seconds)
**Wavenumber Integration** **(SCOOTER)**	Frequency-range-based wavenumber sampling Tabular form of reflection coefficient	Very good Low frequency and short range (Error < 2 dB)	High (thousands of seconds) Proportional to the frequency and the range
**PE** **(RAM)**	Use the Split-step Fourier Algorithm Solving wave equation Boundary scattering not dealt with	Very good for short range (Error < 2 dB)	High (thousands of seconds)
**SAM**	Frequency-independent ray tracing Model-based incorporation of boundary influences Phase shifts carefully dealt with	Good (Error < 5 dB)	Medium (a few seconds)
